# KIS, a target of SOX4, regulates the ID1-mediated enhancement of β-catenin to facilitate lung adenocarcinoma cell proliferation and metastasis

**DOI:** 10.1007/s00432-024-05853-9

**Published:** 2024-07-25

**Authors:** Jing-Xia Chang, Meng Zhang, Li-Li Lou, He-Ying Chu, Hua-Qi Wang

**Affiliations:** Department of Respiratory and Critical Care Medicine, The First Affiliated Hospital of Zhengzhou University, No. 1, Jianshe East Road, Zhengzhou, Henan Province 450000 P.R. China

**Keywords:** KIS, Lung adenocarcinoma, SOX4, ID1, Β-catenin pathway

## Abstract

**Purpose:**

Kinase interacting with stathmin (KIS) is a serine/threonine kinase involved in RNA processing and protein phosphorylation. Increasing evidence has suggested its involvement in cancer progression. The aim of this study was to investigate the role of KIS in the development of lung adenocarcinoma (LUAD). Dual luciferase assay was used to explore the relationship between KIS and SOX4, and its effect on ID1/β-catenin pathway.

**Methods:**

Real-time qPCR and western blot were used to assess the levels of KIS and other factors. Cell proliferation, migration, and invasion were monitored, and xenograft animal model were established to investigate the biological functions of KIS in vitro and in vivo.

**Results:**

In the present study, KIS was found to be highly expressed in LUAD tissues and cell lines. KIS accelerated the proliferative, migratory and invasive abilities of LUAD cells in vitro, and promoted the growth of LUAD in a mouse tumor xenograft model in vivo. Mechanistically, KIS activated the β-catenin signaling pathway by modulating the inhibitor of DNA binding 1 (ID1) and was transcriptionally regulated by SOX4 in LUAD cells.

**Conclusion:**

KIS, a target of SOX4, regulates the ID1-mediated enhancement of β-catenin to facilitate LUAD cell invasion and metastasis.

**Supplementary Information:**

The online version contains supplementary material available at 10.1007/s00432-024-05853-9.

## Introduction

Lung cancer (LC) is one of the cancers with a high incidence and mortality rate. It is estimated that ~ 2.20 million new cases and 1.79 million deaths occur each year due to LC (Thai et al. 2021). LC is classified into non-small cell lung cancer (NSCLC) and small cell lung cancer (SCLC) according to cytological types, with NSCLC accounting for ~ 85% of the diagnosed cases (Denisenko et al. 2018; Hutchinson et al. 2019). Lung adenocarcinoma (LUAD) is the most common subtype of NSCLC (Duma et al. 2019). Patients with LUAD usually have no obvious respiratory symptoms, and thus they are not diagnosed until the disease is at an advanced stage; these patients thus face a high mortality rate (Li et al. 2018; Spella and Stathopoulos 2021). Currently, the main treatment methods for LUAD include chemotherapy, targeted therapies and the use of immune checkpoint inhibitors (Tartour and Zitvogel 2013). With the development of molecular pathology, key genes associated with cancer progression are regarded as targets for targeted therapy (Jin et al. 2020). Although some have achieved good efficacy, the numbers of patients who achieve clinical benefits from this treatment remain limited (Jin et al. 2020; Song et al. 2021; Zuo et al. 2020). It is therefore imperative to identify more effective indicators for the molecular pathology of LUAD.

Kinase interacting with stathmin (KIS) is a serine (Ser, S)/threonine (Thr, T) kinase whose C-terminal domain contains a U2AF homology motif (UHM)(Francone et al. 2010). It can interact with splicing factors, such as SF1 and SF3b155 through the UHM motif (Manceau et al. 2008). Recently, the role of KIS in cancer progression has been reported. An abnormally elevated activity of KIS may be associated with some aspects of tumor development in different types of human cancer, such as pancreatic and ovarian cancer (Grant et al. 2018; Katchman et al. 2017; Wang et al. 2018). Previous studies have suggested roles of KIS in proliferation, migration and invasion of tumor cells (Gao et al. 2022; Xu et al. 2021). KIS knockdown strongly suppresses gastric cancer cell proliferation and invasion (Feng et al. 2020). However, the expression, function and mechanisms of action of KIS in LUAD remain unclear. Based on data from the UALCAN database (Ualcan.path.uab.edu/analysis), it has been found that KIS expression in LUAD is significantly upregulated, suggesting that it plays a role as a tumor promoter.

The inhibitor of DNA binding 1 (ID1) is one of the ID proteins. It is located on human chromosome 20q11 (Mathew et al. 1995). ID1 has been widely studied, and is mainly related to cell senescence, proliferation, survival and tumorigenesis(Suh et al. 2008; Tang et al. 2002). Studies have shown that ID1 promotes the proliferation, migration and invasion of NSCLC cells (Bhattacharya et al. 2010; Li et al. 2017a). It has been shown that the downregulation of ID1 suppresses the malignant phenotype of hepatocellular carcinoma by inhibiting the β-catenin pathway (Chen et al. 2021; Yin et al. 2017). Through the GSE121733 dataset from the Gene Expression Omnibus (GEO) database (https://www.ncbi.nlm.nih.gov/geo/), it has been observed that ID1 expression is significantly downregulated in KIS siRNA-transfected liver cancer cells, indicating ID1 as a potential downstream effector molecule of KIS in liver cancer; however, whether ID1 is regulated by KIS in LUAD remains to be determined.

Sex-determining region Y (Sry)-box-containing (SOX) transcription factors play key developmental roles in the majority of tissues and organs (Sarkar and Hochedlinger 2013). The SOX family includes total of 20 members, and the family is divided into nine subgroups that range from SOXA to SOXH (She and Yang 2015) SOX4 is one of the members widely studied in relation to cancer (Tiwari et al. 2013; Vervoort et al. 2018a). The *SOX4* gene is often amplified and overexpressed in > 20 types of malignancies, including cancers of the lung (Friedman et al. 2004), prostate (Liu et al. 2006), bladder (Aaboe et al. 2006) and breast (Cancer Genome Atlas, 2012). A number of studies have demonstrated that SOX4 knockdown reduces the tumorigenesis and metastasis of cancer cells (Bilir et al. 2013; Vervoort et al. 2018b; Zhang et al. 2012a). In lung cancer, SOX4 also exhibits functional oncogenic properties (Medina et al. 2009). Various miRNAs in NSCLC cells can target SOX4 and inhibit migration and invasion, as well as epithelial-to-mesenchymal transition in cancer cells (Li et al. 2015; Tang et al. 2017), further demonstrating that SOX4 is a complete regulator of malignancies. By predicting that there may be a binding between SOX4 and *KIS* promoter regions, it was hypothesized that SOX4 may transcriptionally influence *KIS* expression.

To the best of our knowledge, the SOX4/KIS/ID1 axis has not been previously reported in lung adenocarcinoma, lung squamous cell carcinoma or other tumors. Based on the aforementioned findings, in the present study, it was hypothesized that KIS may play an oncogenic role in LUAD progression by modulating ID1, and KIS is a potential target of SOX4. The results of the present study provide new insight into the role of KIS in LUAD, which may aid future research.

## Materials and methods

### Bioinformatics analysis

To explore differentially expressed genes (DEGs) between LUAD and normal tissues, gene expression data were obtained from the UALCAN website based on The Cancer Genome Atlas (TCGA) database (http://ualcan.path.uab.edu/analysis.html) and the Gene Expression Omnibus (GEO) database (GSE32863 and GSE40419) (https://www.ncbi.nlm.nih.gov/gds/). The GSE32863 was a dataset of gene expression beadchip for 58 lung adenocarcinoma and 58 adjacent non-tumor lung fresh frozen tissues and the GSE40419 was a dataset of RNA-Seq for 87 lung adenocarcinomas and 77 adjacent normal tissues. DEG analysis of tumor vs. normal tissue from TCGA database and the GEO database was performed using the R package edgeR and the GEO2R online analysis tool, respectively. Genes that met the criteria log_2_|fold change| >0.5 and *P* < 0.01, were considered to be significant DEGs. The results of the intersection of the up- and downregulated genes between TCGA and the GEO datasets are presented in a Venn diagram through using the R package Venn Diagram. Gene Ontology (GO) and Kyoto Encyclopedia of Genes and Genomes (KEGG) pathway enrichment analysis of overlapping DEGs was conducted using the R software ‘cluster Profiler’ package. In addition, Kaplan-Meier Plotter database (https://kmplot.com) was used to analyze the effect of KIS expression on overall survival of LUAD patients.

### Tumor specimens

A total of 30 pairs of fresh tissue samples and paracancerous samples from patients with LUAD who had not received chemoradiotherapy were collected. All patients provided written informed consent to participate in the research. The study methodologies conformed to the standards set by the Declaration of Helsinki and were approved by the Ethical Review Board of the First Affiliated Hospital of Zhengzhou University, Zhengzhou, China.

### Cell lines and cell culture

A total of six LUAD cell lines (cat. no. iCell-h011, A549; cat. no. iCell-h155, NCI-H1650; cat. no. iCell-h156, NCI-H1975; cat. no. iCell-h068, HCC-827; cat. no. iCell-h159, NCI-H358 and cat. no. iCell-h153, NCI-H1299) and an immortalized normal human bronchial epithelial cell line (cat. no. iCell-h023, BEAS-2B) were purchased from iCell Bioscience Inc. and STR profiling was used for authentication. The 293T cells were purchased from Shanghai Zhong Qiao Xin Zhou Biotechnology Co., Ltd. The A549 cells were maintained in F-12 K medium (Wuhan Servicebio Technology Co., Ltd.) containing 10% fetal bovine serum (FBS; Tianhang Bio), while the remaining five LUAD cell lines were grown in Roswell Park Memorial Institute1640 medium (RPMI-1640; Beijing Solarbio Science & Technology Co., Ltd.) containing 10% FBS. The BEAS-2B and 293T cells were maintained in Dulbecco’s modified Eagle’s medium (DMEM; Wuhan Servicebio Technology Co., Ltd.) containing 10% FBS. All cells were maintained in a 5% CO_2_ incubator at 37˚C.

### Reverse transcription-quantitative polymerase chain reaction (RT-qPCR)

The relative RNA levels of genes were assessed using RT-qPCR. Total RNA was isolated using TRIzol reagent (BioTeke Corporation). The concentration of RNA was determined using a UV spectrophotometer NANO 2000 (Thermo Fisher Scientific, Inc.). The obtained RNA was reverse transcribed using BeyoRT II M-MLV reverse transcriptase (Beyotime Institute of Biotechnology). qPCR was performed on an ExicyclerTM 96 (Bioneer) using 2X Taq PCR MasterMix and SYBR-Green (Beijing Solarbio Science & Technology Co., Ltd.) under the following thermocycling conditions: Initial denaturation at 94˚C for 5 min, followed by 40 cycles at 94˚C for 20 s, 60˚C for 30 s and 72˚C for 40 s for denaturation, annealing and elongation. Information regarding primers is presented in Table 1. The relative RNA levels of genes were quantified using the 2^−ΔΔCq^ method(Livak and Schmittgen 2001).

**Table 1:** Information of the primers for qPCR

Primer name	Sequence	Primer length	Product length	Gene ID
KIS F KIS R ID1 F ID1 R c-Myc F c-Myc R Axin2 F Axin2 R SOX4 F SOX4 R	TGTGGCAGGTACAGAGC GCAACAGACAGCGTGAT CGACATGAACGGCTGTTACTC CTCCAACTGAAGGTCCCTGAT ACACCCTTCTCCCTTCG CCGCTCCACATACAGTCC ACCGTGGTTGGCTTGTC TCTACACTGCTGTCCGTCAT GCTGGGCAAACGCTGGAA GCCACCGACCTTGTCTCCCT	17 17 21 21 17 18 17 20 18 20	267 124 179 224 205	NM_175866.5 NM_002165.4 NM_002467.6 NM_004655.4 NM_003107.3

### Western blot analysis

Total protein was extracted from the cells and isolated using RIPA lysis buffer (Beyotime Institute of Biotechnology). Protein quantification was performed using the BCA protein concentration determination kit (Beyotime Institute of Biotechnology). A total of 15–30 µg protein samples were separated by sodium dodecyl sulfate-polyacrylamide gel electrophoresis (SDS‐PAGE) (Beyotime Institute of Biotechnology) on 8%, 10% and 15% gels and then transferred to polyvinylidene fluoride (PVDF) membranes (Thermo Fisher Scientific, Inc.). Subsequently, the membranes were incubated with primary antibodies at 4˚C overnight and secondary antibodies at 37˚C for 40 min, respectively. The antibodies used were as follows: anti-KIS (cat. no. 11624-1-AP; 1:1,000; Proteintech Group, Inc.), anti-PCNA (cat. no. A12427; 1:1,000), anti-CDK4 (cat. no. A11136; 1:1,000), anti-matrix metalloproteinase (MMP)-9 (cat. no. A11147; 1:1,000), anti-MMP-2 (cat. no. A6247; 1:1,000) (all from ABclonal Biotech Co., Ltd.), anti-ID1 (cat. no. ab283650; 1:1,000; Abcam), anti-active β-catenin (cat. no. #33,893; 1:500), anti-c-Myc (cat. no. #18,583; 1:1,000), anti-axis inhibition protein 2 (Axin2; cat. no. #2151; 1:1,000) (all from Cell Signaling Technology, Inc.), anti-SOX4 (cat. no. A10717; 1:500; ABclonal Biotech Co., Ltd.), anti-β-actin (cat. no. 60008-1-Ig; 1:2,000), goat anti-rabbit IgG-HRP (cat. no. SA00001-2; 1:10,000) and goat anti-mouse IgG-HRP (cat. no. SA00001-1; 1:10,000) (all from Proteintech Group, Inc.). The signals were detected using enhanced chemiluminescence (ECL; Shanghai 7 sea biotech Co., Ltd.) and analyzed using a gel imaging system (Gel-Pro-Analyzer software) (WD-9413B, Beijing Liuyi Biotechnology Co., Ltd.).

### Lentivirus production and stable cell line selection

Two LUAD cell lines with a moderate KIS expression (NCI-H1650 and HCC-827) were selected for analyses. The lentivirus particles (Lv-KIS, Lv-Vector Lv-shRNA1-KIS, Lv-shRNA2-KIS and Lv-shNC) were obtained from Wanleibio Co., Ltd. The sequences of shRNAs were as follows: shRNA1-KIS: 5’-ccgGGATGTCAGTGTTTCGGAATTttcaagagaAATTCCGAAACACTGACATCCttttt-3’; shRNA2-KIS: 5’-ccgAGATGTTGTAGAAGATGTAAAttcaagagaTTTACATCTTCTACAACATCTttttt-3’; shNC: 5’-ccgTTCTCCGAACGTGTCACGTttcaagagaACGTGACACGTTCGGAGAAttttt-3’. Briefly, 2.5 µg plasmid or 75 pmol shRNA fragments were cotransfected with lipofectamine 3000 (Thermo Fisher Scientific, Inc.) into 293T cells. After transfection for 48 h at 37˚C, lentivirus particles were harvested. Two LUAD cell lines were infected with Lv-KIS to overexpress KIS and with Lv-shRNA1-KIS/Lv-shRNA2-KIS to knockdown KIS. Stable cell lines were obtained followed by puromycin selection. The efficiency of infection was examined using RT-qPCR and western blot analysis.

### Cell proliferation assay

In order to examine cell proliferation, cell suspensions (5 × 10^3^ cells/well) were seeded in a 96-well plate and cultured in an incubator with 5% CO_2_ at 37˚C. After the cells were cultured for 0, 24, 48 and 72 h, 10 µl Cell Counting Kit-8 (CCK-8) solution (Beijing Solarbio Science & Technology Co., Ltd.) were added to each well. The optical density value of the cells was determined using a microplate reader (BioTek Instruments, Inc.) at 450 nm.

### Colony formation assay

The cells were seeded in petri dishes at a density of 400 cells per dish and cultured in an incubator with 5% CO_2_ at 37˚C. Following incubation at 37˚C for 2 weeks, the cells formed visible cell colonies. The colonies were then fixed with 4% paraformaldehyde (Shanghai Aladdin Biochemical Technology Co., Ltd.) at room temperature for 15 min and stained with Giemsa (Beijing Leagene Biotechnology Co., Ltd.) (Leagene, China) at room temperature for 5 min. The rate of colony formation was calculated.

### Transwell assay

To evaluate cell migration, the LUAD cells were blown off the culture plate and dispersed as single cells in serum-free culture medium. A total of 200 µL cell suspension was then added to the upper chamber of the Transwell (Corning, Inc.). The lower chamber was filled with 800 µL culture medium containing 10% FBS. After culturing in an incubator with 5% CO_2_ and saturated humidity at 37˚C for 24 h, the cells that migrated to the lower chamber were fixed with 4% paraformaldehyde (Shanghai Aladdin Biochemical Technology Co., Ltd.) for 15 min at room temperature. The cells were then washed twice with PBS, stained with crystal violet (Amresco LLC) at room temperature for 2 min and the residual crystal violet solution was washed away with distilled water. Finally, the migrated cells from five random fields were counted under an inverted microscope (Olympus Corporation). To evaluate cell invasion, the upper chamber was precoated with diluted Matrigel (Corning, Inc.). The remaining procedures were the same as those aforementioned for the Transwell migration assay.

### Xenograft animal model

Male BALB/c nude mice (6 weeks old, 16 ± 1 g) were obtained from Jiangsu Huachuang Xinnuo Pharmaceutical Technology Co., Ltd. Mice were provided with free access to food and water in an environment with a 12-h light/dark cycle, a temperature of 22 ± 1˚C and 45–55% humidity. Following 1 week of adaptive feeding, the mice were randomly divided into 4 groups: shNC, shKIS, vector and KIS group. Stable KIS-overexpressing, low-expressing NCI-H1650 cells or control cells (1 × 10^6^ cells in 0.1 mL serum-free medium) were subcutaneously injected into the right axilla of the mice. Animal health and behavior were monitored daily and the tumor volume was monitored every 5 days over a 25-day period. The tumor volume was calculated as follows: ½ (L × W^2^), where L is the length and W is the tumor’s width. Animals were sacrificed by inhalation of CO_2_ at a chamber displacement rate of 30% volume/min. Then the tumors were separated and weighed. The experiments were approved by the Institutional Animal Care and Use Committee of the First Affiliated Hospital of Zhengzhou University.

### Immunohistochemistry

Tumor tissues were fixed with 4% paraformaldehyde at room temperature, dehydrated, embedded in paraffin and sectioned at a thickness of 5 μm. The sections were immunohistochemically stained using anti-Ki67 (cat. no. AF0198; 1:100; Affinity Biosciences) and anti-KIS (cat. no. 11624-1-AP; 1:100; Proteintech Group, Inc.) antibodies. As the secondary antibody, goat anti-rabbit IgG-HRP (cat. no. **#**31,460; 1:500; Thermo Fisher Scientific, Inc.) was used. Staining was performed at room temperature using 3,3-diaminobenzidine (DAB; Fuzhou Maixin Biotech Co., Ltd.) for 10 s and counterstained with hematoxylin (Beijing Solarbio Science & Technology Co., Ltd.) for 3 min.

### Luciferase assay

TopFlash or FopFlash (Beyotime Institute of Biotechnology) and pGMLR-TK luciferase reporter vectors (Beyotime Institute of Biotechnology) were co-transfected into the cells. The cells were cultured in an incubator with 5% CO_2_ at 37˚C for 48 h. The cell suspension was centrifuged at 1000 rpm for 5 min at 4˚C and washed twice with PBS, and then 250 µL cell lysis buffer was added. Following the instructions provided with the luciferase test kit (Nanjing KeyGen Biotech Co., Ltd.), the luciferase activities of cell samples were examined.

Through prediction using the JASPAR database (https://jaspar.genereg.net/), the binding of the SOX4 and KIS promoter was found. In order to detect the binding of the SOX4 and KIS promoter, the KIS promoter fragment was constructed into Firefly luciferase reporter vector and then Firefly luciferase reporter vector and SOX4 overexpression plasmid (1 µg) were co-transfected with lipofectamine 3000 (Thermo Fisher Scientific, Inc.) into 293T cells. After transfection for 48 h, luciferase activity was determined as described above, compared with Renilla luciferase activity.

### Cell transfection

Upon reaching 70% confluency, the cells were transfected with 75 pmol siNC or ID1-siRNA, 2.5 µg SOX4 overexpression plasmid (Ov-SOX4) or control vector using lipofectamine 3000 (Thermo Fisher Scientific, Inc.). The target sequences of siRNAs were as follows: ID1-siRNA: 5’-CAAUGAUCACCGACUGAAATT-3’; siNC: 5’-UUCUCCGAACGUGUCACGUTT-3’.

### Oligonucleotide pull-down assay

The binding of the KIS promoter to SOX4 protein in the NCI-H1650 and HCC-827 cells was verified using the DNA pulldown lit (Guangzhou BersinBio Biological Co., Ltd.) according the manufacturer’s protocol.

### Statistical analyses

All experiments in the present study were performed in triplicate. Data are expressed as the mean ± standard deviation (SD). GraphPad Prism 8.0.2 software was used to analyze the data and draw graphs. Differences between two groups were analyzed using the paired or unpaired Student’s t-test. Differences between multiple groups was analyzed using one-way ANOVA, followed by Tukey’s multiple comparisons test. A P-value < 0.05 was considered to indicate a statistically significant difference.

## Results

### Identification and functional enrichment analysis of DEGs

**Fig. 1:**
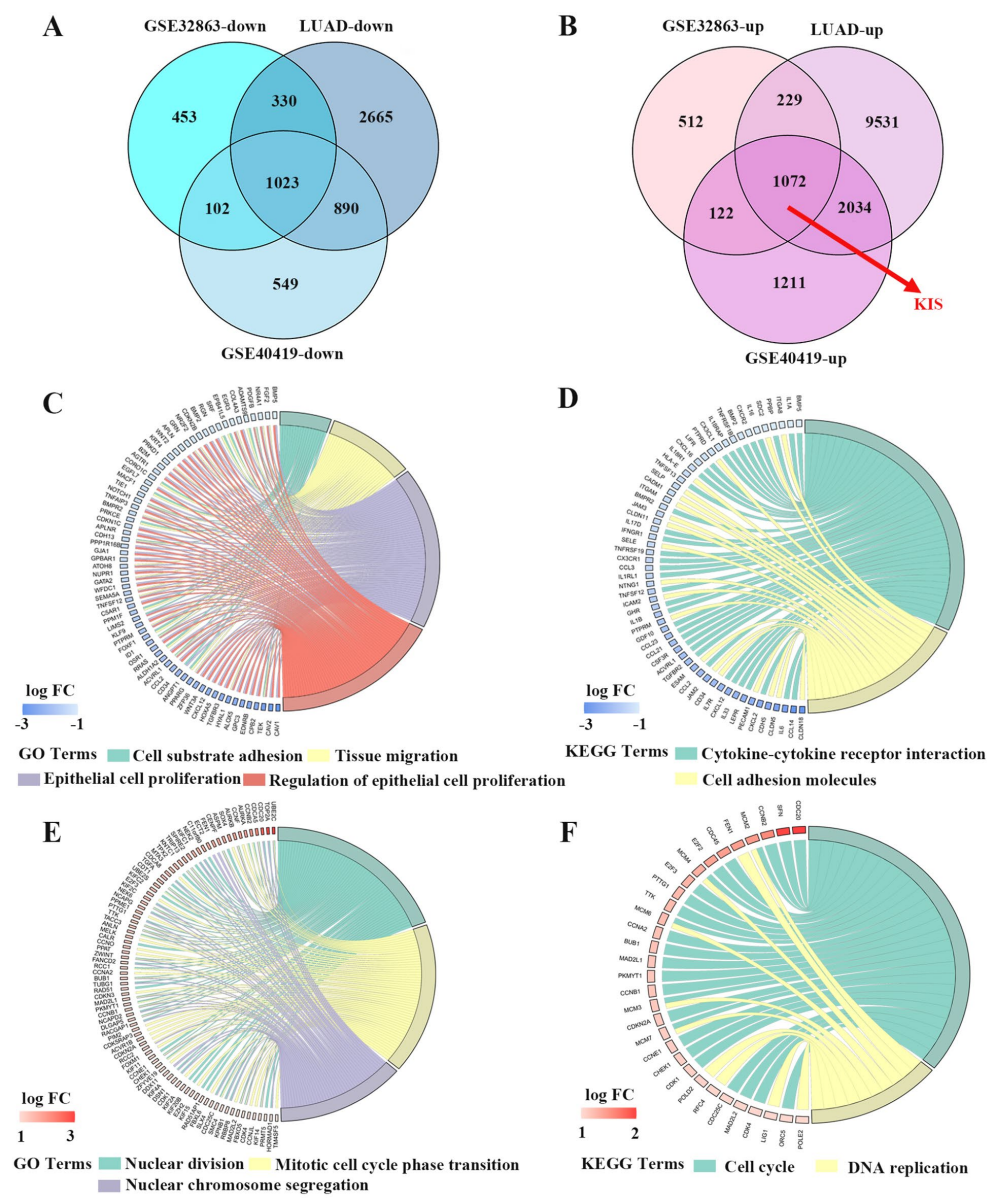
Bioinformatics analysis of DEGs in different gene microarrays of LUAD. **(A** and **B)** A Venn diagram of the **(A)** downregulated and **(B)** upregulated DEGs overlapping between the TCGA LUAD dataset (LUAD) and GEO datasets (GSE32863 and GSE40419). (C and D) The cycle graph of downregulated DEGs enriched in **(C)** GO functional and **(D)** KEGG pathways. **(E** and **F)** The cycle graph of upregulated DEGs enriched in **(E)** GO functional and **(F)** KEGG pathways. The different colored lines indicate different GO terms and KEGG pathways. DEGs, differentially expressed genes; LUAD, lung adenocarcinoma; FC, fold change; GO, Gene Ontology; KEGG, Kyoto Encyclopedia of Genes and Genomes

To identify DEGs between tumor and normal tissues, the present study used TCGA and the GEO databases. A Venn diagram was obtained by the intersection of all up- or downregulated genes in TCGA and the GEO databases. Among these DEGs that passed the screening criteria, 1,023 were significantly downregulated (Fig. 1A) and 1,072 were upregulated (Fig. 1B). KIS was one of the upregulated DEGs and we found that high KIS expression was associated with lower overall survival in LUAD patients through Kaplan-Meier Plotter database (Fig. S1)(Nagy et al. 2021). Besides, we were very interested in the role of KIS in LUAD based on its reports in common cancer diseases including gastric(Feng et al. 2020), liver (Wei et al. 2019) and colorectal (Xu et al. 2021) cancer. Therefore, KIS was selected for further analysis. GO functional and KEGG pathway enrichment analyses of the overlapping DEGs were performed to investigate the role of DEGs in LUAD at a functional level (Fig. S4). The results revealed that the enriched GO terms, including cell substrate adhesion, tissue migration, epithelial cell proliferation and the regulation of epithelial cell proliferation (Fig. 1C), and the enriched KEGG terms, including cytokine-cytokine receptor interaction and cell adhesion molecule (Fig. 1D) were found in the downregulated overlapping DEGs. For the upregulated overlapping DEGs, the enriched GO terms, including nuclear division, mitotic cell cycle phase transition and nuclear chromosome segregation (Fig. 1E), and the enriched KEGG terms, including cell cycle, DNA replication (Fig. 1F) were found.

### KIS is highly expressed in LUAD tissues and cell lines

**Fig. 2:**
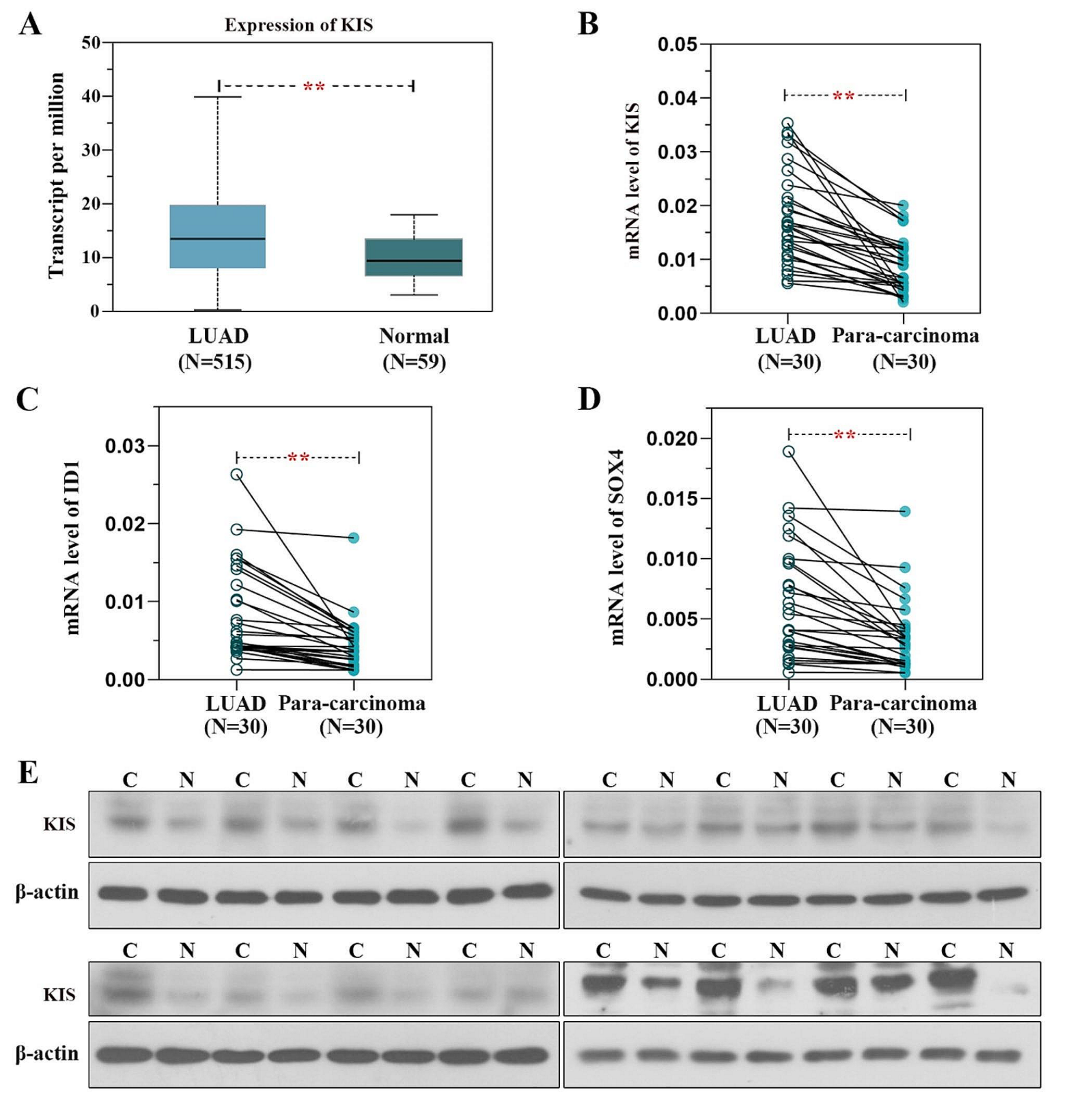
KIS is highly expressed in LUAD tissues and LUAD cell lines. **(A)** KIS mRNA expression in LUAD tissues and normal tissues was analyzed using the UALCAN website based on TCGA database. **(B-D)** The mRNA levels of KIS, ID1 and SOX4 were detected in LUAD tissues from patients who did not receive chemoradiotherapy and paracancerous samples using reverse transcription-quantitative PCR. **(E)** The protein levels of KIS were detected LUAD tissues from patients who did not receive chemoradiotherapy and paracancerous samples using western blot analysis (part of the data). **P < 0.01, compared with normal tissues or paracancerous samples. KIS, Kinase interacting with stathmin; LUAD, lung adenocarcinoma; TCGA, The Cancer Genome Atlas; ID1, inhibitor of DNA binding 1; SOX4, (Sry)-box-containing 4

The mRNA expression of KIS in LUAD and normal tissues was retrieved using the UALCAN website based on TCGA database (Fig. 2A). As demonstrated by the findings, the mRNA expression of KIS was significantly upregulated in LUAD tissues compared with normal tissues, according to the database. To validate this discovery, 30 pairs of fresh tissue samples and paracancerous samples were collected from patients with LUAD who had not received chemoradiotherapy, and the mRNA levels of KIS were detected using RT-qPCR. The results revealed that the mRNA levels of KIS were enhanced in LUAD tissues in comparison with paracancerous tissues (Fig. 2B). As was expected, the mRNA levels of ID1 and SOX4 were also increased (Fig. 2C and D). In addition, similar findings were obtained for the protein levels of KIS in LUAD tissues in comparison with paracancerous tissues (Fig. 2E). Subsequently, the expression of KIS was analyzed in several human LUAD cell lines and a bronchus epithelium cell line using western blot analysis (Fig. S2A and B). As illustrated in figure, the expression of KIS was significantly higher in cancer cells than in normal cells.

### KIS facilitates the proliferation of LUAD cells

**Fig. 3:**
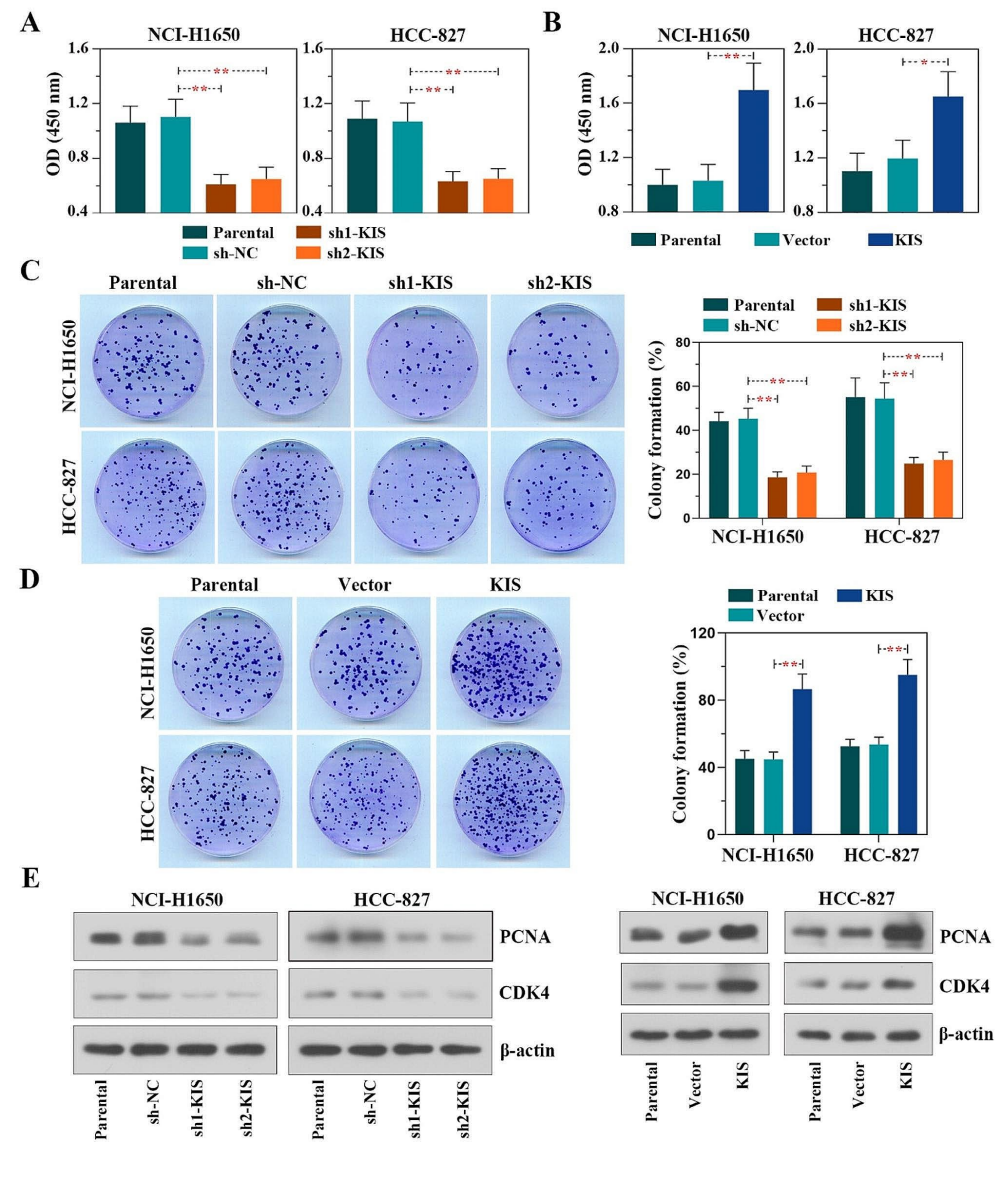
KIS facilitates the proliferation of lung adenocarcinoma cells. **(A** and **B)** The Cell Counting Kit-8 was used to monitor cell proliferation at 72 h following KIS knockdown or overexpression. **(C** and **D)** Colony formation assay of cellular proliferation was conducted following KIS knockdown or overexpression. **(E)** The protein levels of PCNA and CDK4 were detected using western blot analysis in NCI-H1650 (left panel) and HCC-827 (right panel) cells following KIS knockdown or overexpression. *P < 0.05 and **P < 0.01, compared with shNC group or vector group. KIS, kinase interacting with stathmin; PCNA, proliferating cell nuclear antigen; CDK4, cyclin-dependent kinase 4

KIS was successfully knocked down or overexpressed by lentiviral infection in both NCI-H1650 (Fig. S3, left panel) and HCC-827 (Fig. S3, right panel) cells. The results of CCK-8 assay revealed that both the KIS knockdown groups (sh1-KIS, sh2-KIS) exhibited a significantly lower proliferation rate compared with the control group (sh-NC) (Fig. 3A). By contrast, the overexpression group (KIS) exhibited a significantly increased LUAD cell proliferation rate in (Fig. 3B). Consistently, the cell proliferation rate decreased or increased with the knockdown or overexpression of KIS, respectively in both types of cells, as demonstrated using colony formation assay (Fig. 3C and D). Furthermore, western blot analysis revealed that the expression of proliferation-associated proteins, including PCNA and CDK4 decreased/increased significantly with KIS knockdown/overexpression in these two types of cells (Fig. 3E). All the aforementioned results demonstrated that KIS promoted the proliferation of LUAD cells.

### KIS accelerates the migration and invasion of LUAD cells

**Fig. 4:**
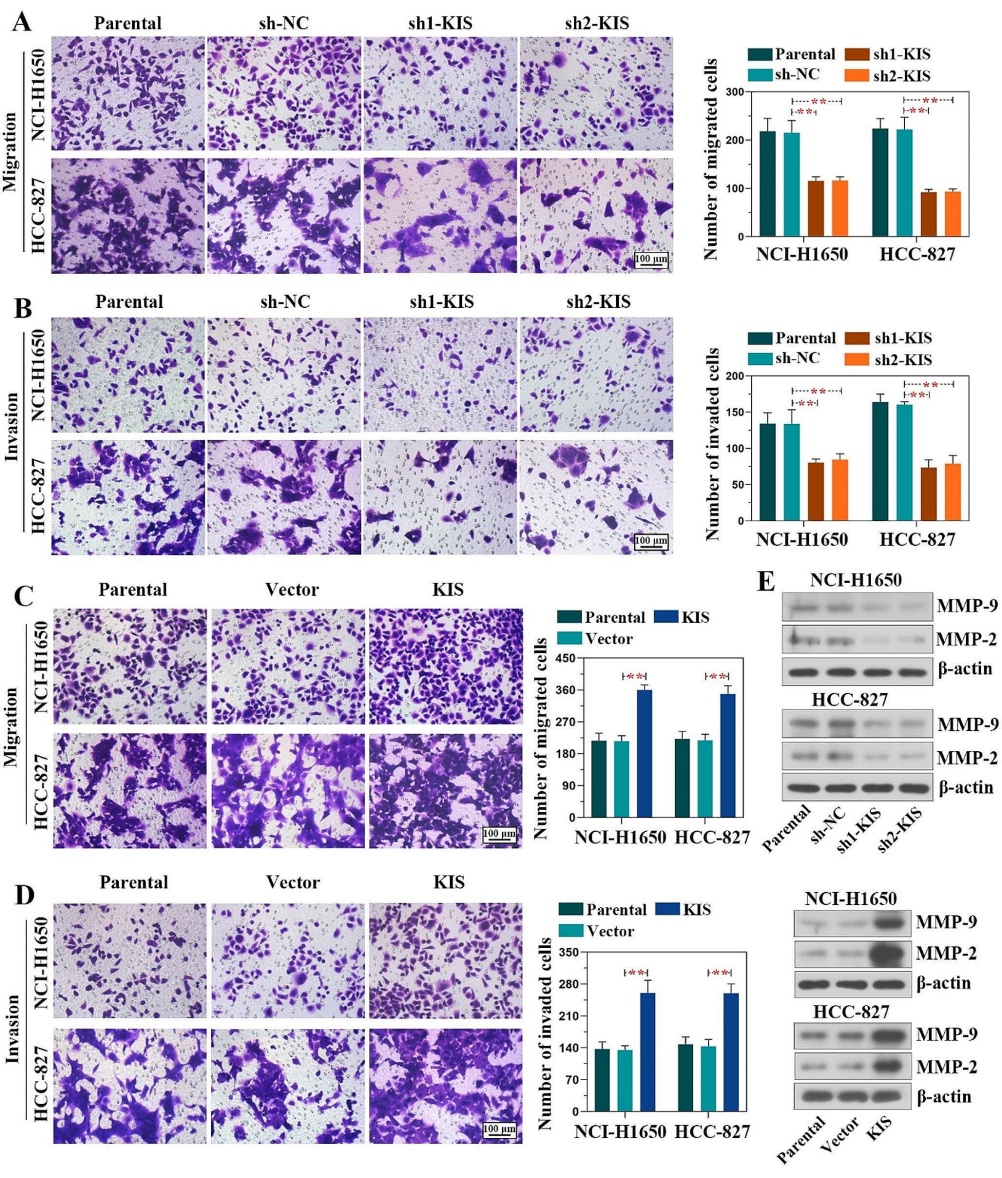
KIS facilitates the migration and invasion of lung adenocarcinoma cells. **(A** and **B)** Images of migratory (upper panel) and invasive (lower panel) cells following KIS knockdown were captured at 24 h. Cell migratoin and invastoin were examined using Transwell assay (scale bar, 100 μm). The graph on the right panel indicates the quantified numbers of migratory and invasive cells. **(C** and **D)** The migratory (upper panel) and invasive (lower panel) capacities were evaluated at 24 h following KIS overexpression (scale bar, 100 μm). **(E)** The protein levels of MMP-9 and MMP-2 were detected using western blot analysis in NCI-H1650 and HCC-827 cells following KIS knockdown or overexpression. ^**^P < 0.01, compared with shNC group or vector group. KIS, kinase interacting with stathmin; MMP, matrix metalloproteinase

The effects of KIS on LUAD cell migration and invasion were investigated using Transwell assays. The results revealed that the knockdown of KIS significantly inhibited (Fig. 4A and B), whereas the overexpression of KIS accelerated the migratory and invasive abilities of LUAD cells (Fig. 4C and D). The protein expression of MMP in LUAD cells was detected using western blot analysis to explore the mechanisms involved in the regulatory effects of KIS on the migration and invasion of LUAD cells (Fig. 4E). The findings suggested that KIS knockdown negatively regulated MMP-2 and MMP-9 expression in LUAD cells, whereas KIS overexpression exerted an opposite regulatory effect. On the whole, these results confirmed that KIS promoted the migration and invasion of LUAD cells.

### KIS promotes tumor growth of LUAD in a nude mouse tumor xenograft model

**Fig. 5:**
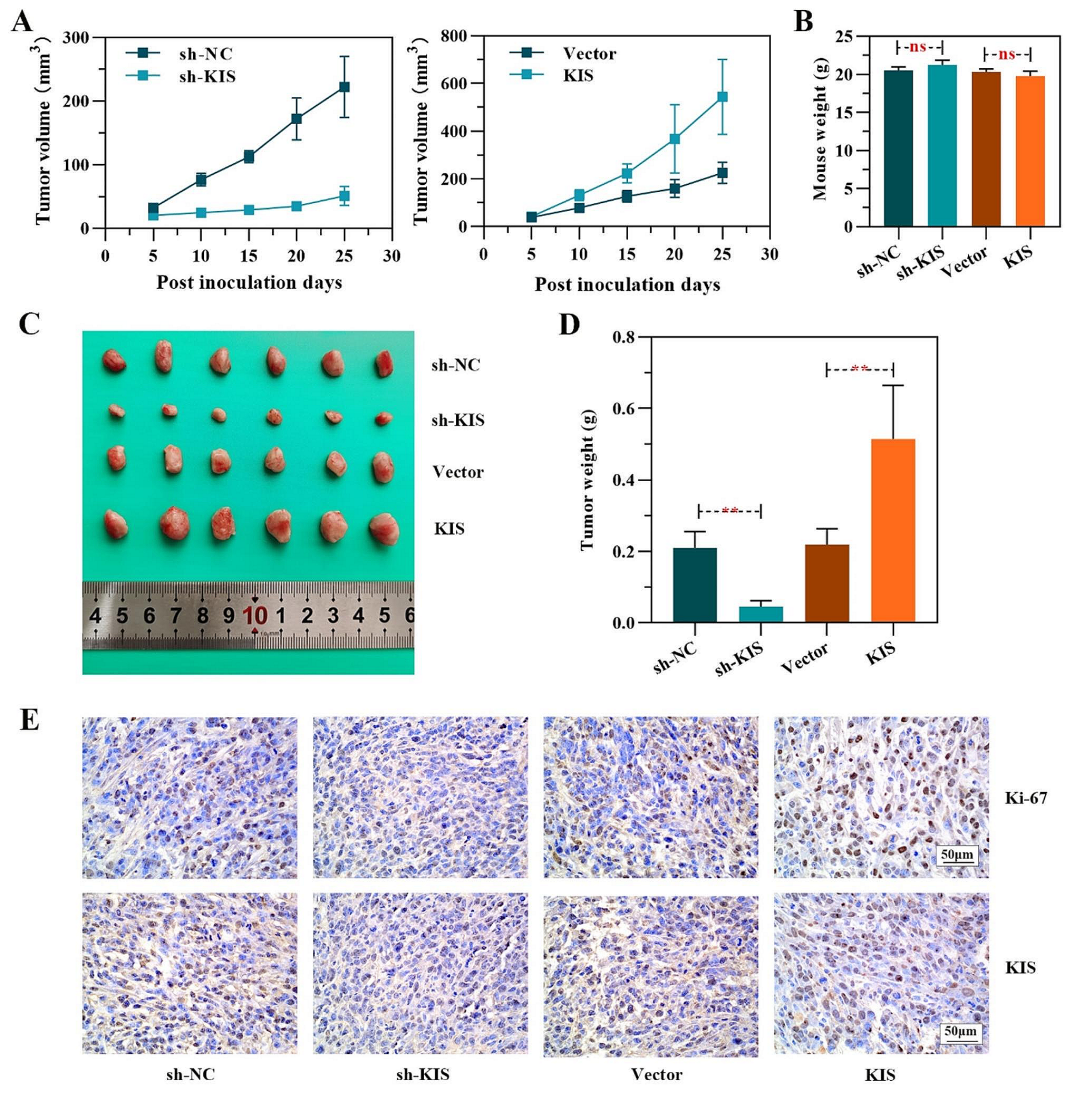
Role of KIS in the tumor growth of NCI-H1650 cells. **(A)** The tumor growth curve of NCI-H1650 cells following KIS knockdown (left panel) or overexpression (right panel) in tumor xenografts from nude mice. **(B) Weight of nude mice after tumor formation. (C)** Tumors from mice injected with NCI-H1650 cells subjected to KIS knockdown or overexpression and the corresponding control. **(D) Weight of tumors derived from NCI-H1650 cells subjected to KIS knockdown or overexpression. (E)** Immunohistochemistry of Ki67 and KIS in tumor tissues of mice. ^**^P < 0.01, compared with shNC group or vector group. KIS, kinase interacting with stathmin

To investigate the tumor-promoting effects of KIS on LUAD cells in vivo, the LUAD cells in which KIS was knocked down/overexpressed and the corresponding control cells were injected into nude mice. The results revealed that there was no significant difference in mouse weight compared to the control group, and tumor progression was significantly inhibited in the mice inoculated with cells in which KIS was knocked down, whereas it was promoted in the mice inoculated with KIS-overexpressing cells as compared with the controls (Fig. 5A-D). Immunohistochemical analysis further demonstrated that the expression of the cell proliferation markers, Ki67 and KIS, was higher/lower in the mice inoculated with cells subjected to KIS overexpression/knockdown as compared with the controls (Fig. 5E).

### KIS activates the ID1-mediated β-catenin signaling pathway

**Fig. 6:**
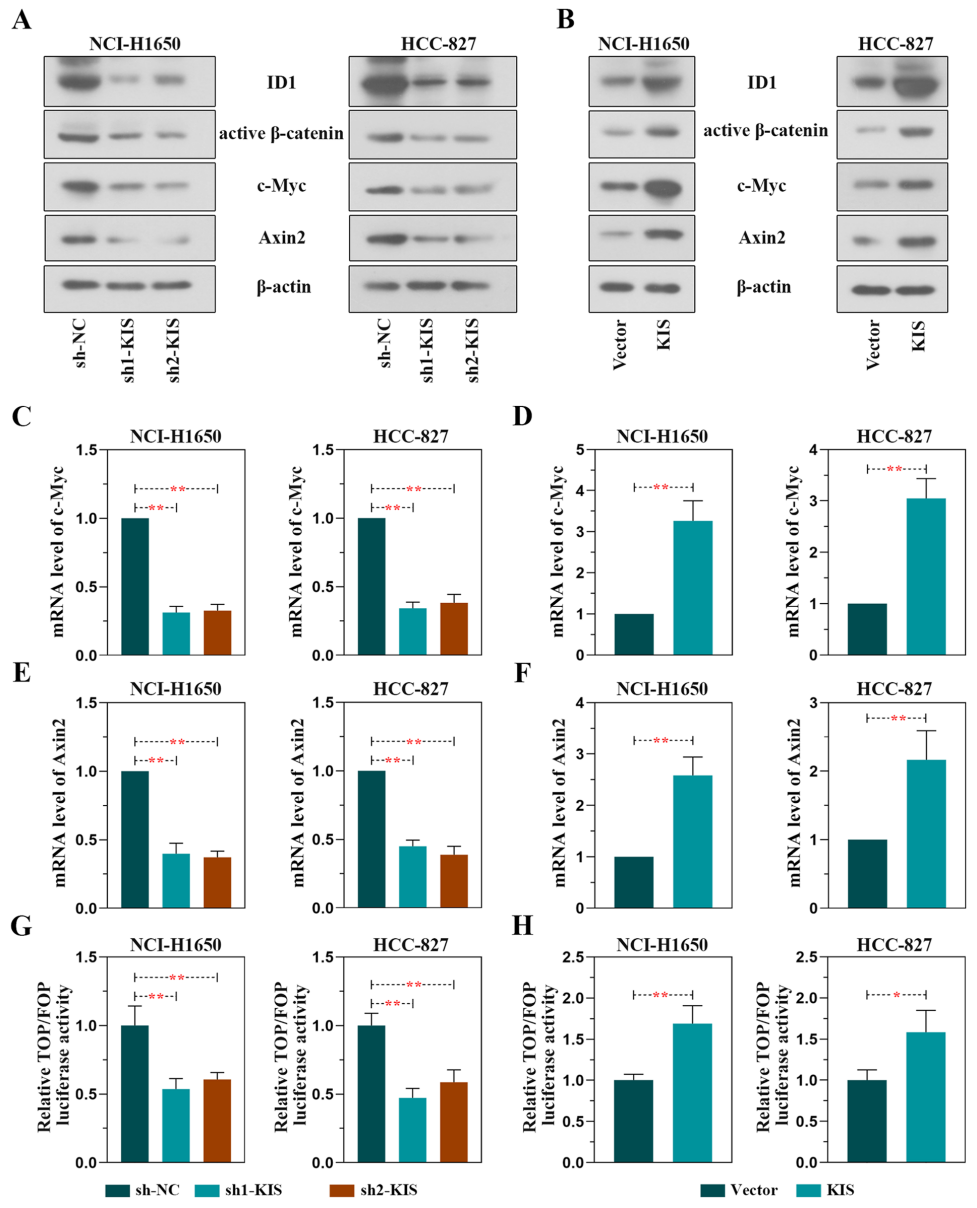
KIS facilitates the activation of the ID1-mediated β-catenin signaling pathway. NCI-H1650 and HCC-827 cells were used to perform the experiments. **(A** and **B)** The protein levels of ID1, active β-catenin, c-Myc and Axin2 were detected following KIS knockdown or overexpression. (C-F) The mRNA levels of c-Myc and Axin2 were measured following KIS knockdown or overexpression. **(G** and **H)** TOP/FOP flash assay revealed the luciferase activity of β-catenin following KIS knockdown or overexpression. ^*^P < 0.05 and ^**^P < 0.01, compared with shNC group or vector group. KIS, kinase interacting with stathmin; ID1, inhibitor of DNA binding 1; Axin2, axis inhibition protein 2

As ID1 was identified as a potential downstream effector molecule of KIS via analysis of KIS siRNA-transfected liver cancer cells in the GSE121733 dataset, the ID1 and its related β-catenin pathway was further investigated in the present study. The levels of ID1 and the pathway-related proteins, active β-catenin, c-Myc and Axin2, were examined in LUAD cells in which KIS was knocked down or overexpressed (Fig. 6A and B). The results revealed that the protein levels of ID1, active β-catenin, c-Myc and Axin2 were significantly decreased in the KIS knockdown groups (Fig. 6A), whereas they were markedly increased in the overexpression group (Fig. 6B). Simultaneously, the results of RT-qPCR demonstrated that the silencing of KIS led to the downregulation of the mRNA levels of c-Myc and Axin2, two downstream factors of β-catenin (Fig. 6C and E); however, the overexpression of KIS led to the upregulation of the levels of these two factors (Fig. 6D and F). Furthermore, results of dual luciferase assay revealed that β-catenin activity was significantly lower in the cells in which KIS was knocked down (Fig. 6G), whereas it was higher in the cells overexpressing KIS (Fig. 6H). Collectively, results suggested that KIS the activated ID1-mediated β-catenin signaling pathway in LUAD.

### KIS promotes LUAD development by modulating ID1

**Fig. 7:**
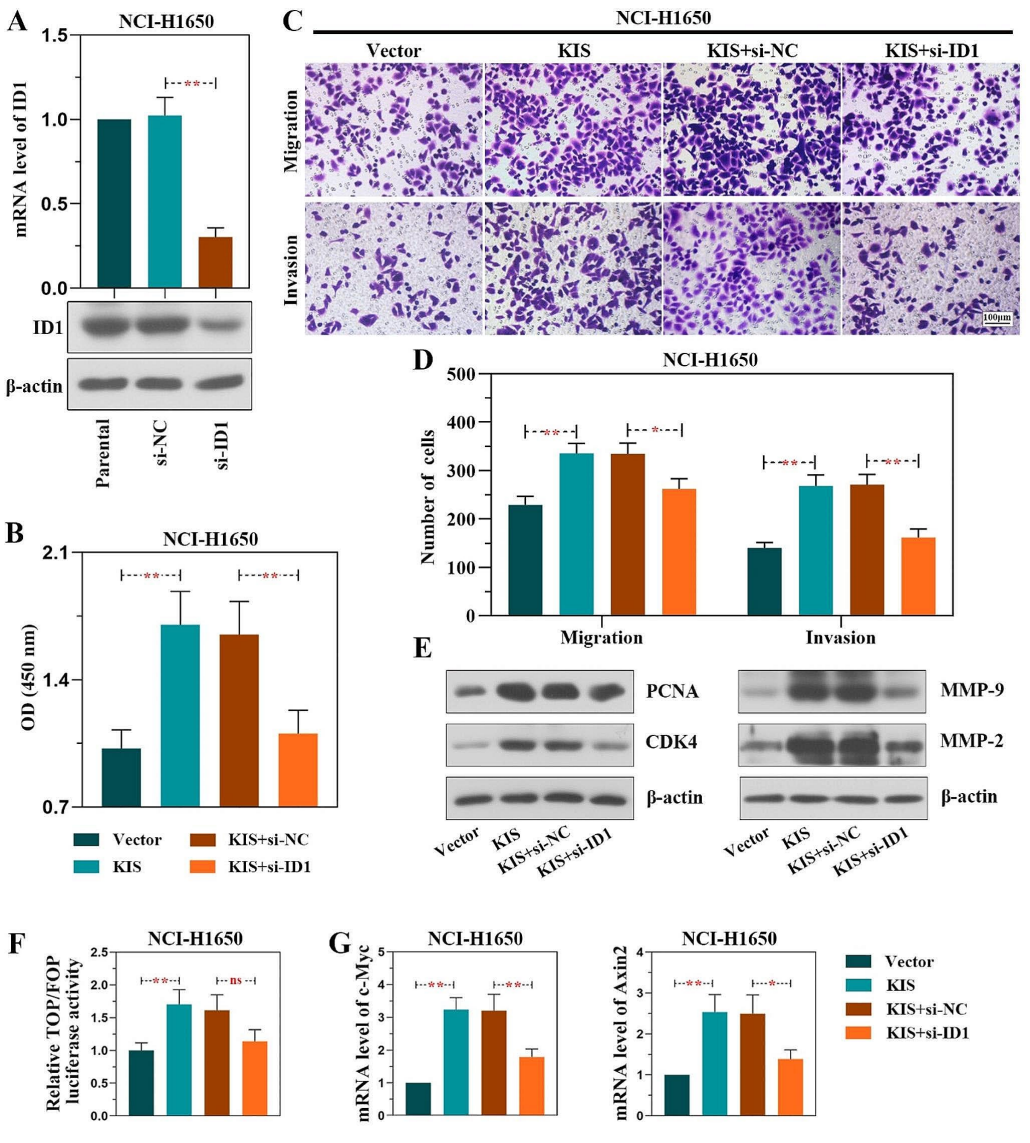
KIS facilitates LUAD development by modulating ID1. **(A)** The transfection efficiency of ID1 shRNA in NCI-H1650 cells was verified at the mRNA and protein level. KIS-overexpressing NCI-H1650 cells were transfected with ID1 siRNA to silence ID1, and these cells were subjected to analyses following 24 h of transfection. **(B)** Cell Counting Kit-8 assay of cellular proliferation. **(C)** The migratory (upper panel) and invasive (lower panel) capacities of cells were evaluated at 24 h following transfection using Transwell assay (scale bar, 100 μm). **(D)** The numbers of migratory and invasive cells were quantified. **(E)** The protein levels of PCNA, CDK4, MMP-9 and MMP-2 were detected using western blot analysis. **(F)** TOP/FOP flash assay revealed the luciferase activity of β-catenin. **(G)** The mRNA levels of c-Myc and Axin2 were measured. ^*^P < 0.05 and ^**^P < 0.01, compared with shNC group, vector group or KIS + si-NC group. KIS, kinase interacting with stathmin; ID1, inhibitor of DNA binding 1; Axin2, axis inhibition protein 2; PCNA, proliferating cell nuclear antigen; CDK4, cyclin-dependent kinase 4; MMP, matrix metalloproteinase

To further evaluate whether ID1 is responsible for the promoting effects of KIS on LUAD progression, the NCI-H1650 cells stably overexpressing KIS were transfected with ID1 siRNA. First, ID1 siRNA (si-ID1) was transfected into blank cells for the detection of the transfection efficiency, and the expression of ID1 was confirmed using RT-qPCR and western blot analysis (Fig. 7A). Subsequently, it was determined whether ID1 knockdown exerted a suppressive effect on the ability of KIS to promote the proliferation, migration and invasion of LUAD cells. Similar to previous results, KIS overexpression promoted the proliferation, migration and invasion of cancer cells. However, KIS-overexpressing cells transfected with ID1 siRNA (KIS + si-ID1) exhibited a significantly lower proliferation, migration and invasion rate compared with the control siRNA cells (KIS + si-NC) in vitro (Fig. 7B-D). Furthermore, the results of western blot analysis of the expression of PCNA, CDK4, MMP-9 and MMP-2 revealed that ID1 knockdown suppressed the proliferation, migration and invasion of KIS-overexpressing cells (Fig. 7E). In addition, KIS overexpression enhanced the luciferase activity of β-catenin, and elevated the mRNA expression of c-Myc and Axin2; however, opposite results were obtained in KIS-overexpressing cells transfected with ID1 siRNA (Fig. 7F and G). These findings thus suggested that the function of KIS in LUAD was partially mediated by ID1.

### KIS is transcriptionally regulated by SOX4 in LUAD cells

**Fig. 8:**
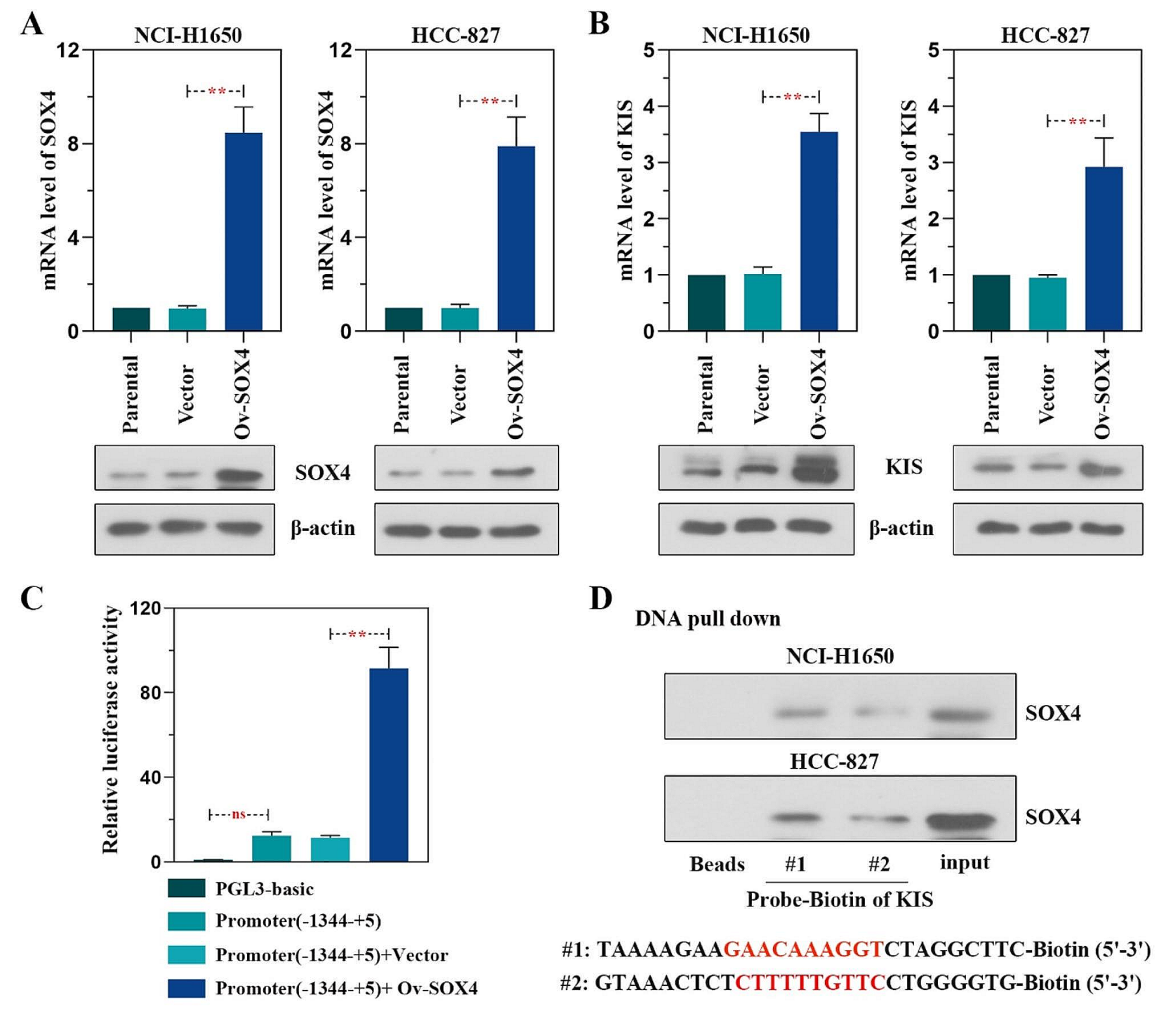
KIS is regulated by SOX4 in lung adenocarcinoma cells. **(A)** The transfection efficiency of SOX4 overexpression was verified in NCI-H1650 and HCC-827 cells at the mRNA and protein level. **(B)** KIS mRNA and protein levels were increased following SOX4 overexpression. **(C)** The effect of SOX4 on KIS-driven promoter activity was explored using a luciferase assay. **(D)** DNA pull-down assay was conducted with KIS biotinylated binding sites #1 and #2 as probes. Western blot analysis revealed that KIS bound to SOX4. ^**^P < 0.01, compared with vector group or Promoter (-1344-+5) + Vector group; ns, not significant, compared with PGL3-basic group. KIS, kinase interacting with stathmin; SOX4, (Sry)-box-containing 4

Through prediction using the JASPAR database (https://jaspar.genereg.net/), binding sites were identified between the *KIS* promoter region and the transcription factor, SOX4, suggesting that *KIS* may be a target of SOX4. To validate this finding, NCI-H1650 and HCC-827 cells were transfected with SOX4 overexpression plasmid. As shown in Fig. 8A, the overexpression of SOX4 was confirmed to be successful by the detection of mRNA and protein expression. Notably, the KIS mRNA and protein levels increased after SOX4 was overexpressed (Fig. 8B). The combination of SOX4 and *KIS* promoter was then detected using luciferase assay. The results demonstrated that the luciferase activity of SOX4 on the *KIS*-driven promoter was significantly promoted (Fig. 8C). DNA pull-down assay and western blot analysis revealed that the *KIS* promoter bound to SOX4 (Fig. 8D). These results thus suggested that KIS was regulated by SOX4 in LUAD cells.

## Discussion

In the present study, with the use of bioinformatics analysis of LUAD DEGs in TCGA database and two datasets from the GEO database, it was found that KIS was an upregulated DEG overlapping in these datasets. GO and KEGG pathway enrichment analyses revealed that these overlapping DEGs were also associated with the division and proliferation of cells, and the cell cycle, which revealed that cell growth, invasion and migration were critical physiological processes leading to the development of LUAD. The oncogenic role of KIS in the progression of several types of cancer has been reported, such as gastric cancer (Feng et al. 2020), hepatocellular carcinoma (Wei et al. 2019) and ovarian cancer(Katchman et al. 2017); however, its expression pattern and functional role in LUAD remain unclear. Similar reports aimed at defining the role of KIS in pancreatic ductal adenocarcinoma (PDAC) have demonstrated that KIS expression is low in non-malignant pancreatic cells and tissues, whereas its expression is significantly increased in several PDAC cell lines and tissues (Luo et al. 2022a). Similar results were obtained in the present study, in that KIS was highly expressed in LUAD tissues and cell lines, suggesting that KIS plays a role in the progression of LUAD. However, a limitation of the present study was that we were unable to obtain sufficient clinical samples to perform an analysis of the association between KIS expression and clinical parameters in patients with LUAD. We mainly explored the mechanism of KIS in LUAD from the cellular perspective.

LUAD cells are characterized by insidious, high infiltration and destructive growth (Yu et al. 2021). In the early stages, tumors often invade the body through lymph or blood vessel (Zhang et al. 2018). Proliferation, migration and invasion are key properties to describe cells. A previous study reported that the the oncogene yes-associated protein (YAP)-dependent induction of KIS supports the proliferation, but not the migration of liver cancer cells. Additionally, KIS induces cell proliferation and cell cycle progression through the phosphorylation of p27^kip1^ in leukemia cells (Nakamura et al. 2008). In the present study, KIS overexpression promoted the proliferation of LUAD cells, whereas KIS knockdown exerted the opposite effects. However, as opposed to its role in liver cancer cells, KIS promoted the migration and invasion of LUAD cells. A possible explanation for this may be that the targets of KIS differ among diverse types of cancer cells.

ID proteins are a subgroup of the basic helix-loop-helix (bHLH) proteins which lack the basic DNA-binding region (Meng et al. 2020). ID proteins function as dominant negative regulators of bHLH transcription factors by heterodimerization with other bHLH factors, and inhibiting their binding to DNA (Zhao et al. 2020). ID1 has been reported to play a carcinogenic role in a variety of tumors. In tumor cells, increased levels of ID1 are associated with a poorly differentiated and aggressive phenotype (Schindl et al. 2003; Schoppmann et al. 2003), and ID1 affects cell survival and metastasis by regulating multiple pathways. For example, the activation of mitogen-activated protein kinase (MAPK) signaling has been demonstrated as a mechanism of ID1-induced cancer cell proliferation (Ling et al. 2002). Shin et al. found that ID1 was able to regulate the expression of Wingless and INT-1 (Wnt)/β-catenin signal transduction regulators(Shin et al. 2011). Another study demonstrated that the activation of Wnt-β-catenin signaling led to the accumulation of β-catenin in the nucleus, which occurs in > 80% of colorectal cancer cases (Wanitsuwan et al. 2008). In more than half of cancer cases, including breast, colorectal, melanoma and leukemia, β-catenin accumulates in the nucleus or cytoplasm (Damsky et al. 2011; Gekas et al. 2016; Khramtsov et al. 2010; Kobayashi et al. 2000). In addition, β-catenin promotes tumor progression by inhibiting T-cell responses (Hong et al. 2015). β-catenin activity is influenced by binding factors that affect its stability, cellular localization and transcriptional activity (Shang et al. 2017). Axin2 and proto-oncogene c-Myc are two representative target genes of the β-catenin pathway (Rennoll et al. 2014). As previously reported, ID1 induces c-Myc activation through the Wnt/β-catenin pathway, thereby promoting G6PD transcription, and then activating the pentose phosphate pathway, resulting in chemoresistance to oxaliplatin in hepatocellular carcinoma (Yin et al. 2017). The Wnt/β-catenin signaling pathway is a complex pathway that regulates the expression of key developmental genes by regulating the level of β-catenin (Cheng et al. 2019). β-catenin is a signal converter in cells, whose abnormal regulation can lead to early carcinogenesis (Zhang and Wang 2020). However, to the best of our knowledge, to date, there is no available evidence to reveal the effects of KIS on the ID1/β-catenin pathway in LUAD cells. Therefore, to demonstrate this effect, the present study measured the mRNA and protein expression levels of ID1, c-Myc and Axin2, and the activity of active β-catenin. In the present study, KIS overexpression increased both the levels of three factors and the activity of active β-catenin, while KIS knockdown exerted the opposite effect. These results indicated that KIS promoted the ID1-mediated β-catenin signaling pathway. The present study then investigated whether the effects of KIS on LUAD progression are mediated via ID1. The results demonstrated that KIS-overexpressing cells transfected with ID1 siRNA reversed the promoting effects of KIS overexpression on the proliferation, migration and invasion of LUAD cells. Moreover, KIS-overexpressing cells transfected with ID1 siRNA exhibited a suppressed activity of β-catenin, as well as decreased mRNA levels of c-Myc and Axin2. Therefore, it was confirmed that KIS facilitated LUAD progression by modulating ID1.

SOX4, a member of the SOX transcription factor family, is also upregulated in a number of human malignancies(Dai et al. 2017). Our previous research has suggested that miR-363-3p targets NEDD9 and SOX4 to the inhibit migration, invasion and epithelial-mesenchymal transition of NSCLC cells (Chang et al. 2020). In another study, shRNA was used to specifically knock down SOX4 in the Xuanwei female lung cancer cell line (XWLC-05), and experiments using nude mice revealed that this led to increased apoptosis, and decreased cell proliferation and metastasis (Zhou et al. 2015). These findings suggest that SOX4 is a factor involved in LC. Notably, the results reported in the study by Zhang et al. demonstrated that SOX4 promoted breast cancer growth and metastasis, and upregulated the transcriptional level of one of the cytokines (CXCR7) by binding to the *CXCR7* promoter (Zhang et al. 2020). Moreover, binding may exist between SOX4 and KIS promoter regions through predictions from the JASPAR database. The results of the present study confirmed this hypothesis and demonstrated that *KIS* expression was upregulated by SOX4 in LUAD cells. In other words, *KIS* is a downstream gene of SOX4.

In clinical trials, high levels of SOX4 protein were positively correlated with status of differentiated degree, clinical stage, T classification, N classification, M classification in NSCLC, and the higher level of SOX4 expression was markedly correlated with poor overall survival in NSCLC patients (Wang et al., 2015). Similar clinical analyses about SOX4 in acute myeloid leukemia (Lu et al. 2017), breast cancer (Zhang et al., 2012b) and bladder carcinoma (Aaboe et al. 2006) have also been reported. In addition, the clinical significance and biological role of ID1 in lung cancer was emphasized (Castañón et al., 2017; Li et al. 2017b; Román et al. 2019). In view of KIS, it was considered to be a novel marker for personalized prediction of pancreatic cancer prognosis (Luo et al., 2022b) and was associated with a mechanism of non-genetic resistance to targeted therapy in melanoma (Smith et al., 2022). According to the above results in this study, the potential role of KIS inhibition is expected to be a new target for LUAD treatment.

**Fig. 9:**
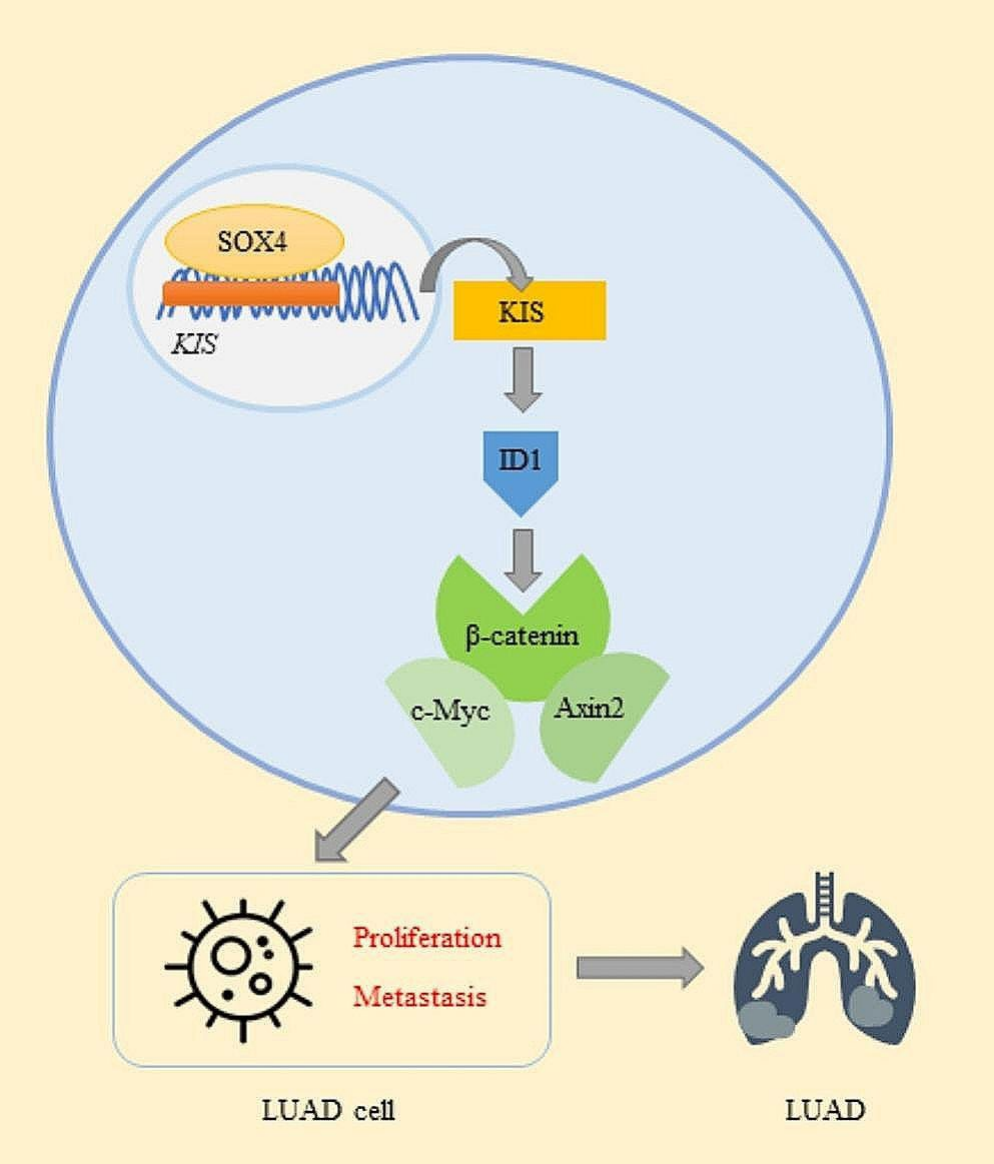
Potential role of KIS in the development of LUAD. In the cancerogenesis of LUAD, KIS is transcriptionally regulated by SOX4 and activates ID1-mediated β-catenin signaling pathway, which in turn promotes the proliferation and metastasis of LUAD cells and accordingly impacts the progression of LUAD. KIS, kinase interacting with stathmin; LUAD, lung adenocarcinoma

In conclusion, the findings of the present study demonstrated that KIS promoted the proliferation, migration and invasion of LUAD cells through the ID1-mediated β-catenin signaling pathway, and was regulated by SOX4 in LUAD cells (Fig. 9). The SOX4/KIS/ID1/β-catenin axis has the potential to function as a therapeutic target in LUAD. KIS inhibitors will be the focus of our future research.

### Electronic supplementary material

Below is the link to the electronic supplementary material.

Supplementary Material 1

## Data Availability

The datasets generated and analyzed during the present study are available from the corresponding author upon reasonable request.
