# Supplementary material for: KIS, a target of SOX4, regulates the ID1-mediated enhancement of β-catenin to facilitate lung adenocarcinoma cell proliferation and metastasis
Source: J Cancer Res Clin Oncol. 2024 Jul 25;150(7):366. doi: 10.1007/s00432-024-05853-9 (PMC11272720; doi:10.1007/s00432-024-05853-9)

**Supplementary figure legends**

**Figure S1.** Survival analyses of KIS mRNA expression levels in LUAD patients. (A) Survival analyses of KIS mRNA expression levels in LUAD based on the Kaplan-Meier Plotter database. Kaplan–Meier survival curves showed significant differences in OS between LUAD patients with high and low KIS mRNA expression levels (HR = 1.55, log-rank *P* = 0.01). KIS, kinase interacting with stathmin; LUAD, lung adenocarcinoma; OS, overall survival.

**Figure S2**. Western blot analysis (A) and corresponding quantification (B) of KIS expression in different human lung adenocarcinoma cells (A549, NCI-H1650, NCI-H1975, HCC-827, NCI-H358 and NCI-H1299) and human bronchial epithelial cells (BEAS-2B). KIS, kinase interacting with stathmin.

**Figure S3**. (A and B) KIS was successfully knocked down or overexpressed using lentivirus infection both in NCI-H1650 (left panel) and HCC-827 (right panel) cells. ^**^*P*<0.01, compared with shNC group or vector group. KIS, kinase interacting with stathmin.

**Figure S4** GO and KEGG pathway enrichment analysis. (A) Top 30 GO enrichment terms of downregulated (left) and upregulated (right) DEGs. (B) Top 30 KEGG pathways of downregulated (left) and upregulated (right) DEGs. DEGs, differentially expressed genes; GO, Gene Ontology; KEGG, Kyoto Encyclopedia of Genes and Genomes.


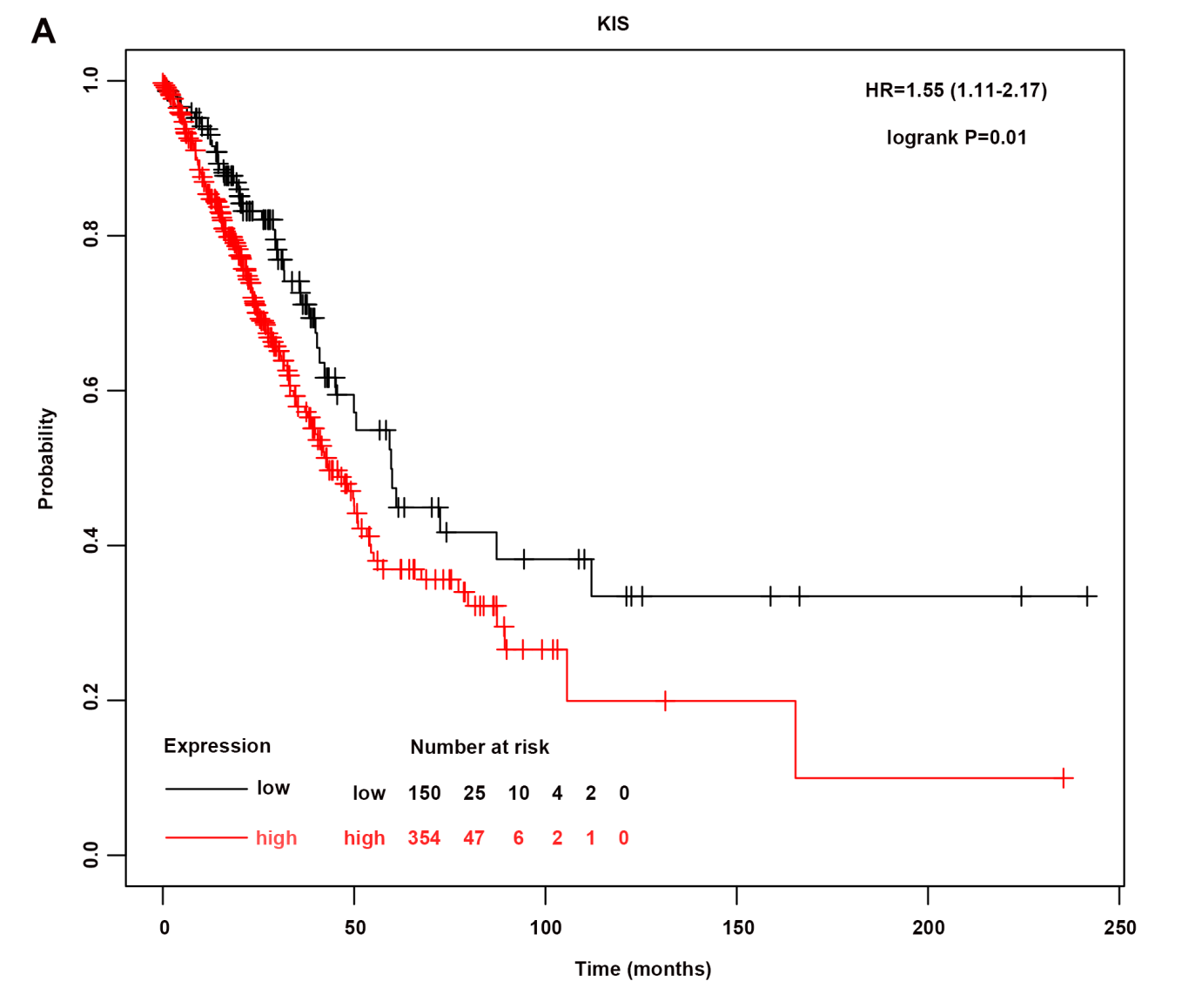


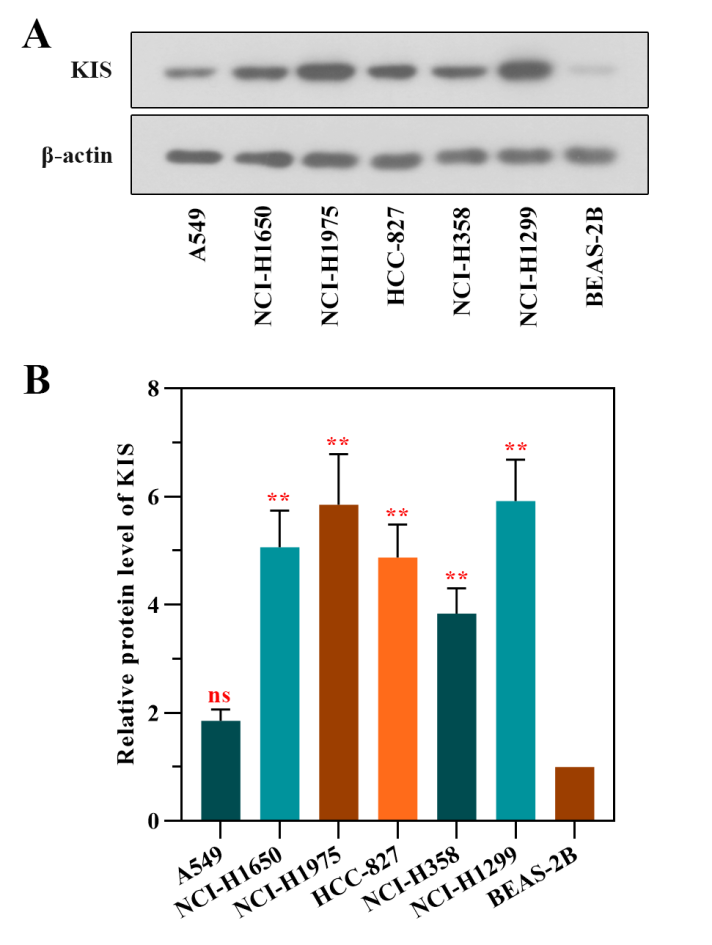


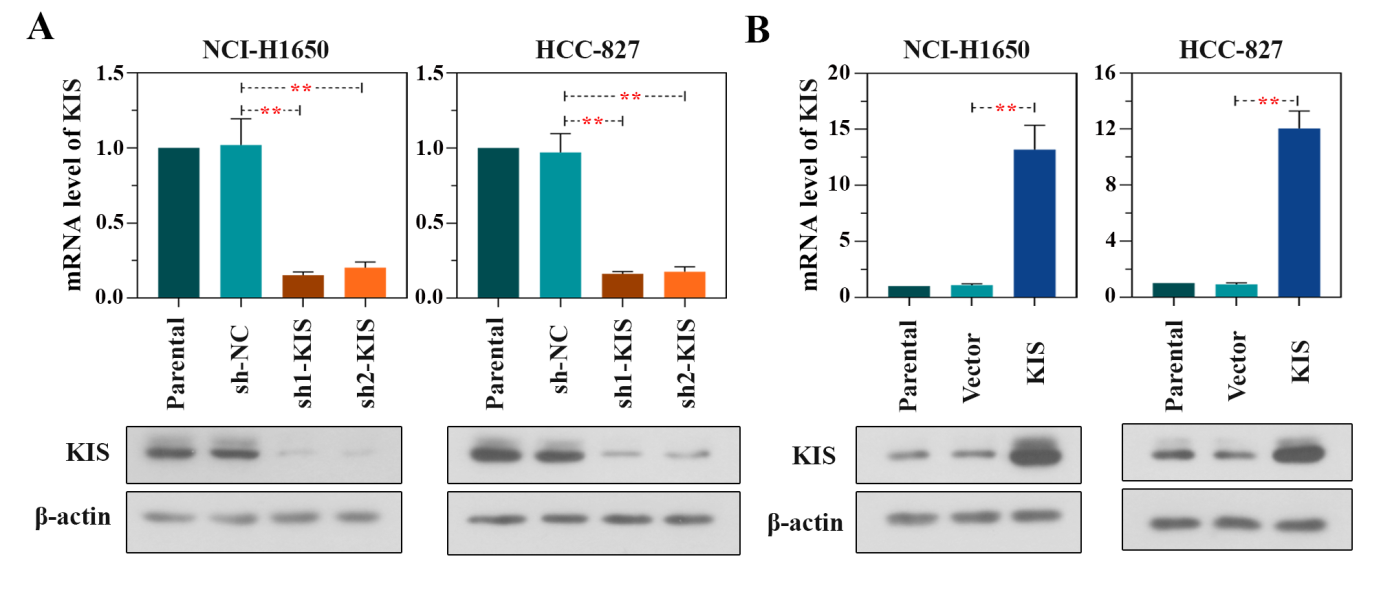


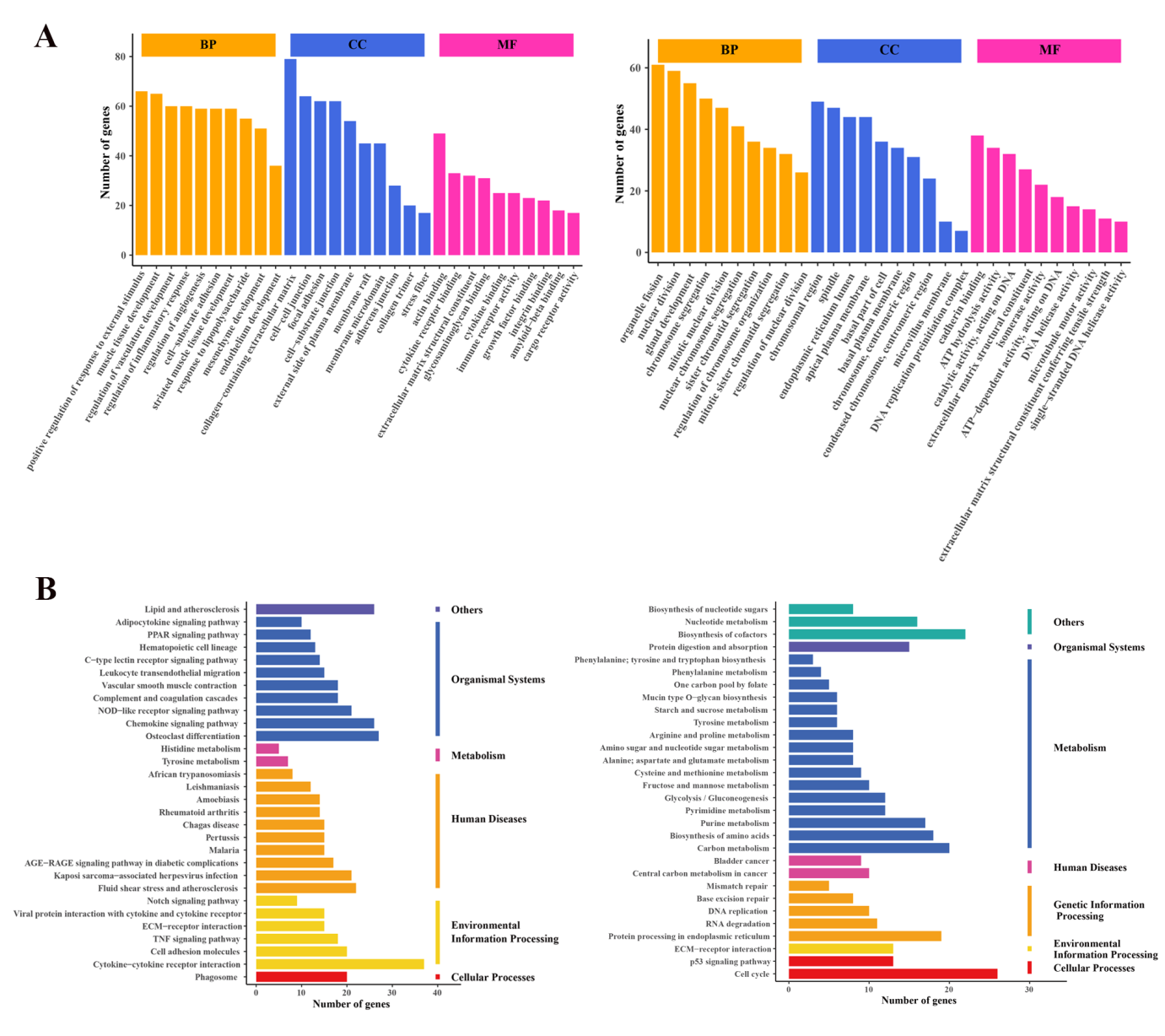

Supplement: Supplementary file 1 — Supplementary Material 1 [file 432_2024_5853_MOESM1_ESM.docx]
